# Supplementary material for: Accuracy of the electronic health record’s problem list in describing multimorbidity in patients with heart failure in the emergency department
Source: PLoS One. 2022 Dec 13;17(12):e0279033. doi: 10.1371/journal.pone.0279033 (PMC9747000; doi:10.1371/journal.pone.0279033)
Supplement: S1 Table — HF = Heart Failure. (PDF) [file pone.0279033.s001.pdf]

S1 Table. Comparison of demographics of full population of ED patients with HF population as compared to selected random sample.

		Complete population of ED patients with HF		Random sample of ED patients with HF	
		**N**	**%**	**N**	**%**
Total		1130	100.0%	200	100.0%
Age					
	65-69	258	22.8%	48	24.0%
	70-74	224	19.8%	27	13.5%
	75-79	195	17.3%	35	17.5%
	80-84	155	13.7%	32	16.0%
	85+	298	26.4%	58	29.0%
Gender					
	Female	582	51.5%	105	52.5%
	Male	547	48.4%	95	47.5%
Race					
	White	765	67.7%	130	65.0%
	Black	311	27.5%	57	28.5%
	Other	54	4.8%	13	6.5%
Ethnicity					
	Non-Hispanic	1095	96.9%	191	95.5%
	Hispanic	31	2.7%	9	4.5%
Primary Payer					
	Medicare	1078	95.4%	191	95.5%
	Non-Medicare	52	4.6%	9	4.5%

HF = Heart Failure
