# Supplementary material for: Accuracy of the electronic health record’s problem list in describing multimorbidity in patients with heart failure in the emergency department
Source: PLoS One. 2022 Dec 13;17(12):e0279033. doi: 10.1371/journal.pone.0279033 (PMC9747000; doi:10.1371/journal.pone.0279033)
Supplement: S2 Table — *Indeterminate kappa due to complete agreement on either positive cases or negative cases resulting in a 0 in the denominator; PABAK = prevalence-adjusted, bias-adjusted kappa. (PDF) [file pone.0279033.s002.pdf]

S2 Table. **Reliability in measuring multimorbidity by structured chart review.**

	No. observed by chart reviewer 1	No. observed by chart reviewer 2	PABAK	
**Elixhauser domain**	**N (%)**	**N (%)**	**k**	**95% CI**
Total	30 (100%)	30 (100%)	-	-
Acquired immune deficiency syndrome	1 (3.33%)	1 (3.33%)	1	1.00-1.00
Alcohol abuse	4 (13.33%)	4 (13.33%)	1	1.00-1.00
Anemias, deficiency	12 (40.00%)	8 (26.67%)	0.73	0.49-0.98
Arthropathies	0 (0%)	1 (3.33%)	*	
Chronic blood loss anemia	0 (0%)	2 (6.67%)	*	
Cancer - Leukemia	1 (3.33%)	1 (3.33%)	1	1.00-1.00
Cancer - Lymphoma	1 (3.33%)	1 (3.33%)	1	1.00-1.00
Cancer, metastatic	0 (0%)	0 (0%)	*	
Cancer - Solid tumor without metastasis, in situ	0 (0%)	1 (3.33%)	*	
Cancer - Solid tumor without metastasis, malignant	3 (10.00%)	2 (6.67%)	0.93	0.81-1.00
Cerebrovascular disease	4 (13.33%)	2 (6.67%)	0.73	0.49-0.98
Coagulopathy	5 (16.67%)	6 (20.00%)	0.8	0.59-1.00
Dementia	3 (10.00%)	4 (13.33%)	0.93	0.81-1.00
Depression	11 (36.67%)	11 (36.67%)	0.87	0.69-1.00
Diabetes with chronic complications	9 (30.00%)	9 (30.00%)	0.87	0.69-1.00
Diabetes without chronic complications	5 (16.67%)	4 (13.33%)	0.67	0.40-0.93
Drug abuse	2 (6.67%)	1 (3.33%)	0.93	0.81-1.00
Hypertension, complicated	1 (3.33%)	2 (6.67%)	0.93	0.81-1.00
Hypertension, uncomplicated	29 (96.67%)	24 (80.00%)	0.67	0.40-0.93
Liver disease, mild	5 (16.67%)	3 (10.00%)	0.87	0.69-1.00
Liver disease, moderate to severe	0 (0%)	0 (0%)	*	
Pulmonary disease, chronic	11 (36.67%)	10 (33.33%)	0.93	0.81-1.00
Neurological disorders affecting movement	3 (10.00%)	2 (6.67%)	0.93	0.81-1.00
Neuro -Other neurological disorders	2 (6.67%)	0 (0%)	*	
Neuro - Seizures and epilepsy	2 (6.67%)	2 (6.67%)	1	1.00-1.00
Obesity	16 (53.33%)	15 (50.00%)	0.8	0.59-1.00
Paralysis	1 (3.33%)	1 (3.33%)	1	1.00-1.00
Peripheral vascular disease	6 (20.00%)	5 (16.67%)	0.8	0.59-1.00
Psychoses	2 (6.67%)	2 (6.67%)	1	1.00-1.00
Pulmonary circulation disease	2 (6.67%)	2 (6.67%)	0.87	0.69-1.00
Renal failure, moderate	5 (16.67%)	6 (20.00%)	0.93	0.81-1.00
Renal failure, severe	2 (6.67%)	2 (6.67%)	1	1.00-1.00
Hypothyroidism	7 (23.33%)	5 (16.67%)	0.87	0.69-1.00
Other thyroid disorders	1 (3.33%)	2 (6.67%)	0.93	0.81-1.00

Peptic ulcer with bleeding	5 (16.67%)	5 (16.67%)	0.73	0.49-0.98
Valvular disease	4 (13.33%)	5 (16.67%)	0.93	0.81-1.00
Weight loss	2 (6.67%)	1 (3.33%)	0.93	0.81-1.00

*Indeterminate kappa due to complete agreement on either positive cases or negative cases resulting in a 0 in the denominator ; PABAK = prevalence-adjusted, bias-adjusted kappa
