# Supplementary material for: Accuracy of the electronic health record’s problem list in describing multimorbidity in patients with heart failure in the emergency department
Source: PLoS One. 2022 Dec 13;17(12):e0279033. doi: 10.1371/journal.pone.0279033 (PMC9747000; doi:10.1371/journal.pone.0279033)
Supplement: S3 Table — EHR = electronic health record; PPV = positive predictive value; NPV = negative predictive value. (PDF) [file pone.0279033.s003.pdf]

S3 Table. Diagnostic accuracy of measuring Elixhauser comorbid domains from the EHR-based problem list for all 37 domains.

	Prevalence by chart review (gold standard)	Sensitivity	Specificity	PPV	NPV
**Elixhauser domain**	**% (N)**	**% (95% CI)**	**% (95% CI)**	**% (95% CI)**	**% (95% CI)**
Acquired immune deficiency syndrome	1.5% (3)	100.0% (29.2%-100.0%)	100.0% (98.1%-100.0%)	100.0% (29.2%-100.0%)	100.0% (98.1%-100.0%)
Alcohol abuse	4.5% (9)	88.9% (51.8%-99.7%)	100.0% (98.1%-100.0%)	100.0% (63.1%-100.0%)	99.5% (97.1%-100.0%)
Anemias, deficiency	33% (66)	69.7% (57.1%-80.4%)	97.8% (93.6%-99.5%)	93.9% (83.1%-98.7%)	86.8% (80.3%-91.7%)
Arthropathies	4.5% (9)	100.0% (66.4%-100.0%)	99.5% (97.1%-100.0%)	90.0% (55.5%-99.7%)	100.0% (98.1%-100.0%)
Cancer - Leukemia	1.5% (3)	100.0% (29.2%-100.0%)	100.0% (98.1%-100.0%)	100.0% (29.2%-100.0%)	100.0% (98.1%-100.0%)
Cancer - Lymphoma	2.5% (5)	80.0% (28.4%-99.5%)	100.0% (98.1%-100.0%)	100.0% (39.8%-100.0%)	99.5% (97.2%-100.0%)
Cancer - Solid tumor without metastasis, in situ	0.5% (1)	100.0% (2.5%-100.0%)	98.5% (95.7%-99.7%)	25.0% (0.6%-80.6%)	100.0% (98.1%-100.0%)
Cancer - Solid tumor without metastasis, malignant	19.5% (39)	76.9% (60.7%-88.9%)	98.8% (95.6%-99.8%)	93.8% (79.2%-99.2%)	94.6% (90.1%-97.5%)
Cancer, metastatic	1.5% (3)	66.7% (9.4%-99.2%)	100.0% (98.1%-100.0%)	100.0% (15.8%-100.0%)	99.5% (97.2%-100.0%)
Cerebrovascular disease	28% (56)	57.1% (43.2%-70.3%)	99.3% (96.2%-100.0%)	97.0% (84.2%-99.9%)	85.6% (79.4%-90.6%)
Chronic blood loss anemia	1% (2)	100.0% (15.8%-100.0%)	97.5% (94.2%-99.2%)	28.6% (3.7%-71.0%)	100.0% (98.1%-100.0%)
Coagulopathy	12.5% (25)	52.0% (31.3%-72.2%)	99.4% (96.9%-100.0%)	92.9% (66.1%-99.8%)	93.5% (89.0%-96.6%)
Dementia	12% (24)	58.3% (36.6%-77.9%)	99.4% (96.9%-100.0%)	93.3% (68.1%-99.8%)	94.6% (90.3%-97.4%)
Depression	27% (54)	51.9% (37.8%-65.7%)	98.6% (95.1%-99.8%)	93.3% (77.9%-99.2%)	84.7% (78.4%-89.8%)
Diabetes with chronic complications	30.5% (61)	85.2% (73.8%-93.0%)	97.8% (93.8%-99.6%)	94.5% (84.9%-98.9%)	93.8% (88.5%-97.1%)

Diabetes without chronic complications	17% (34)	52.9% (35.1%-70.2%)	97.6% (93.9%-99.3%)	81.8% (59.7%-94.8%)	91.0% (85.8%-94.8%)
Drug abuse	3.5% (7)	100.0% (59.0%-100.0%)	100.0% (98.1%-100.0%)	100.0% (59.0%-100.0%)	100.0% (98.1%-100.0%)
Hypertension, complicated	3.5% (7)	85.7% (42.1%-99.6%)	97.4% (94.1%-99.2%)	54.5% (23.4%-83.3%)	99.5% (97.1%-100.0%)
Hypertension, uncomplicated	90% (180)	77.8% (71.0%-83.6%)	100.0% (83.2%-100.0%)	100.0% (97.4%-100.0%)	33.3% (21.7%-46.7%)
Hypothyroidism	23.5% (47)	63.8% (48.5%-77.3%)	100.0% (97.6%-100.0%)	100.0% (88.4%-100.0%)	90.0% (84.5%-94.1%)
Liver disease, mild	5.5% (11)	63.6% (30.8%-89.1%)	99.5% (97.1%-100.0%)	87.5% (47.3%-99.7%)	97.9% (94.8%-99.4%)
Liver disease, moderate to severe	2% (4)	50.0% (6.8%-93.2%)	100.0% (98.1%-100.0%)	100.0% (15.8%-100.0%)	99.0% (96.4%-99.9%)
Neuro - Seizures and epilepsy	3.5% (7)	85.7% (42.1%-99.6%)	99.5% (97.1%-100.0%)	85.7% (42.1%-99.6%)	99.5% (97.1%-100.0%)
Neuro -Other neurological disorders	4.5% (9)	88.9% (51.8%-99.7%)	98.4% (95.5%-99.7%)	72.7% (39.0%-94.0%)	99.5% (97.1%-100.0%)
Neurological disorders affecting movement	6% (12)	75.0% (42.8%-94.5%)	100.0% (98.1%-100.0%)	100.0% (66.4%-100.0%)	98.4% (95.5%-99.7%)
Obesity	42% (84)	28.6% (19.2%-39.5%)	97.4% (92.6%-99.5%)	88.9% (70.8%-97.6%)	65.3% (57.7%-72.4%)
Other thyroid disorders	7% (14)	85.7% (57.2%-98.2%)	99.5% (97.0%-100.0%)	92.3% (64.0%-99.8%)	98.9% (96.2%-99.9%)
Paralysis	1.5% (3)	100.0% (29.2%-100.0%)	99.5% (97.2%-100.0%)	75.0% (19.4%-99.4%)	100.0% (98.1%-100.0%)
Peptic ulcer with bleeding	7% (14)	21.4% (4.7%-50.8%)	99.5% (97.0%-100.0%)	75.0% (19.4%-99.4%)	94.4% (90.2%-97.2%)
Peripheral vascular disease	24% (48)	77.1% (62.7%-88.0%)	96.7% (92.5%-98.9%)	88.1% (74.4%-96.0%)	93.0% (87.9%-96.5%)
Psychoses	1.5% (3)	100.0% (29.2%-100.0%)	97.0% (93.5%-98.9%)	33.3% (7.5%-70.1%)	100.0% (98.1%-100.0%)
Pulmonary circulation disease	13% (26)	73.1% (52.2%-88.4%)	100.0% (97.9%-100.0%)	100.0% (82.4%-100.0%)	96.1% (92.2%-98.4%)
Pulmonary disease, chronic	38% (76)	76.3% (65.2%-85.3%)	99.2% (95.6%-100.0%)	98.3% (90.9%-100.0%)	87.2% (80.6%-92.3%)
Renal failure, moderate	25.5% (51)	68.6% (54.1%-80.9%)	98.7% (95.2%-99.8%)	94.6% (81.8%-99.3%)	90.2% (84.5%-94.3%)

Renal failure, severe	13% (26)	84.6% (65.1%-95.6%)	98.9% (95.9%-99.9%)	91.7% (73.0%-99.0%)	97.7% (94.3%-99.4%)
Valvular disease	26% (52)	80.8% (67.5%-90.4%)	98.6% (95.2%-99.8%)	95.5% (84.5%-99.4%)	93.6% (88.5%-96.9%)
Weight loss	6.5% (13)	69.2% (38.6%-90.9%)	97.9% (94.6%-99.4%)	69.2% (38.6%-90.9%)	97.9% (94.6%-99.4%)

EHR = electronic health record ; PPV = positive predictive value ; NPV = negative predictive value
