# Supplementary material for: Accuracy of the electronic health record’s problem list in describing multimorbidity in patients with heart failure in the emergency department
Source: PLoS One. 2022 Dec 13;17(12):e0279033. doi: 10.1371/journal.pone.0279033 (PMC9747000; doi:10.1371/journal.pone.0279033)

S1 Fig. Flow diagram of selection of patients for the study cohort.

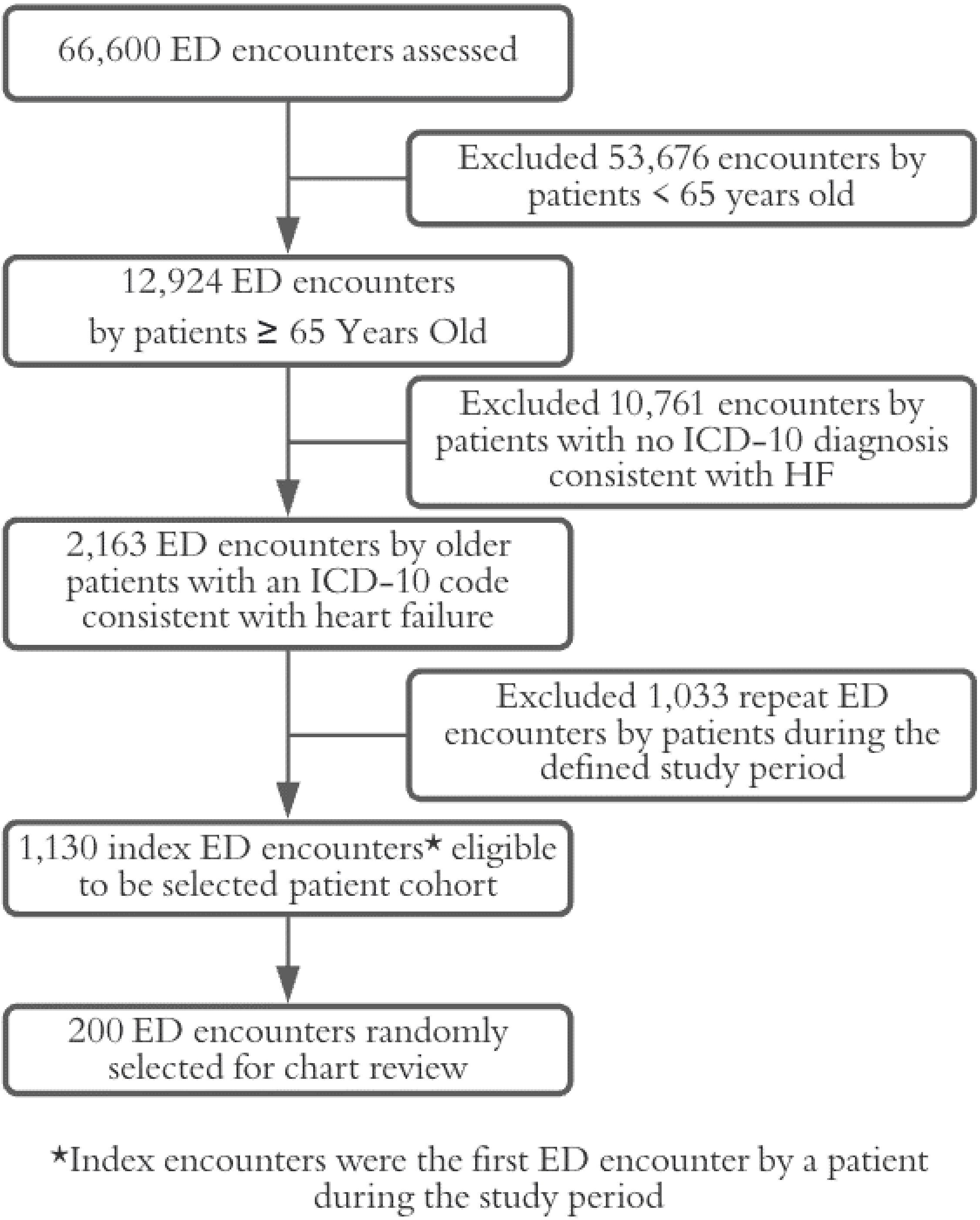

Supplement: S1 Fig — (PDF) [file pone.0279033.s004.pdf]
