# Supplementary material for: Accuracy of the electronic health record’s problem list in describing multimorbidity in patients with heart failure in the emergency department
Source: PLoS One. 2022 Dec 13;17(12):e0279033. doi: 10.1371/journal.pone.0279033 (PMC9747000; doi:10.1371/journal.pone.0279033)
Supplement: Attachment — Submitted filename: Response to reviewers.pdf [file pone.0279033.s008.pdf]

Review Comments to the Author

Please use the space provided to explain your answers to the questions above. You may also include additional comments for the author, including concerns about dual publication, research ethics, or publication ethics. (Please upload your review as an attachment if it exceeds 20,000 characters)

Reviewer #1: Casey et al. present a study to evaluate a multimorbidity ‘problem list’ via electronic health records (EHRs) for patients with heart failure (HF) admitted in emergency departments (ED). The population sample was drawn from patients admitted in the ED of a southeastern US academic medical center. The inclusion criteria were determined on the basis of ICD-10 and demographics. An problem list across multiple disease domains was defined and multimorbidity was measured as gold standard via a 4-steps thorough chart review (plus additional materials if needed, e.g., echocardiography reports) performed by two trained persons and further checked by MD and nurse practitioner. The AHRQ Elixhauser (ICD-10) comorbidity score was calculated by manually reviewing ICD-10 codes. Domain based, and overall concordance indices for the cart review were calculated (i.e., PABAK, ICC), and then PPV and NPV of the gold standard with Elixhauser scores (three summary indices).
Results show a large range of variability in both the concordance indices and the diagnostic accuracy when stratifying by domain. In particular, there was poor capture of obesity and hypertension. The authors conclude that the diagnostic accuracy of the EHR-based multimorbidity problem list for patients with HF in ED.

Overall, the manuscript is well-written and well-structured, with balance among the presented sections.
However, there are concerns about the study rationale/premise, and robustness of analysis for which the conclusive claims or the clinical relevance might be hampered, diminished.

AUTHORS: Thanks for the positive feedback on our writing. We recognize the limitations of the proposed methodology, specifically that 1.7 domains were not identified per patient when using the EHR-based problem list. Although the proposed methodology for measuring multimorbidity has clear limitations, the method does form the basis of a promising foundation which can be built upon by extracting additional data elements (e.g., lab values, radiographic findings) to improve overall accuracy. We have updated the manuscript text to acknowledge this feedback (see page 9-10), which we very much agree with.

Methodological issues:
1. Study sample. First, ED/HF patients would possibly present as a selected population with comorbidity bias in one or more domains; the authors acknowledge that in part, but it may be a major driver. Second, the random sample was 200 over 1,130 eligible patients in the EHR data extraction. Even if chart review might have been too cumbersome for the whole sample, at least comparison with demographics and other descriptive statistics should have been made.

AUTHORS: We have included a new supplemental table that compares the demographics of the 1,130 eligible patients to the 200 patients randomly selected for chart review. We believe the values are comparable across the two groups (reflecting that random selection of charts for review was performed correctly; see Supplemental Table 1).

2. Most of EHR systems nowadays provide automated calculation of comorbidity index, usually the Charlson's. The way in which the calculation is performed can vary, e.g., the time of lookback for finding a chronic condition, or the number of times a condition is diagnosed to confirm, and can be associated to a measurement error, population variance. The Elixhauser calculation presented here might have affected in the same way. The authors should have tried to consider a fully automated score provided by the EHR system.

AUTHORS: We have investigated whether our healthcare system's EHR includes any measures of comorbidity such the Elixhauser or Charlson index. Unfortunately, our current EHR build does not include any measures of comorbidity. Thank you for this suggestion.

3. The moderate PABAK and ICC in certain domains also indicates is substantial variability in clinical determinations, and this happens in parallel with the Elixhauser score determination, likely affecting the resulting diagnostic accuracy.

AUTHORS: We included a discussion on this topic in our limitation section (see page 10). Thank you for this suggestion.

4. Given the continuous scales, a threshold analysis could have been useful (e.g., ROCs).

AUTHORS: We maximized study power by using continuous scales, rather than thresholds. Unfortunately, the idea of multimorbidity measurement as a standalone measurement is still relatively nascent. We are unaware of any consensus agreements on meaningful thresholds that could be used to guide an ROC analysis comparing domain count and Elixhauser index scores. We do appreciate this suggestion, but do not see viable approach to implementing a meaningful ROC analysis.

Reviewer #2

Clinical significance issues:
The work --as is-- stands as a mere exercise to compare imperfect, resource-intensive measures. The authors acknowledge the limitations. Yet, possibly, a predictive-based development based on these preliminary results could provide the translational relevance that does not emerge here.

AUTHORS: See above. We have expanded some language in our manuscript to highlight use of additional structured data elements (such as, laboratory values, radiographic findings) and advanced methods (such as machine-learning) which incorporate unstructured data in capturing the burden of multimorbidity (see page 10).

Reviewer #2: Thank you for a neat study. This would be improved by including:
1. The means of random chart selection.

AUTHORS: We have a included a description and citation on the random sequence generator used to select charts in our methods. Thank you for this suggestion.

2. Assessment of key demographics (which ought to be readily suitable for automated statistical count) between the sample and the full non-excluded population - such as age, gender, ethnicity. This would confirm that the sample was representative in practice.

AUTHORS: See above commentary on inclusion of new Supplemental Table 1.

3. Discussion of the hypothetical savings which could be obtained in ED time if applied as a routine practice - e.g. saved time per patient; patients per day.

AUTHORS: We have expanded on the implications for ED physicians (see page 9), though with the caveats that the EHR-problem list is not a perfect technique. We are not aware of any studies on the exact time ED physicians spend obtaining medical history per patient, and thus are unable to quantify a meaningful hypothetical savings. We do appreciate this suggestion.

Journal Requirements:
When submitting your revision, we need you to address these additional requirements.
1. Please ensure that your manuscript meets PLOS ONE's style requirements, including those for file naming. The PLOS ONE style templates can be found at https://journals.plos.org/plosone/s/file?id=wjVg/PLOSOne_formatting_sample_main_body.pdf and https://journals.plos.org/plosone/s/file?id=ba62/PLOSOne_formatting_sample_title_authors_affiliations.pdf

AUTHORS: We have reviewed the guidelines and updated the body of text accordingly.

2. Please provide additional details regarding participant consent. In the ethics statement in the Methods and online submission information, please ensure that you have specified (1) whether consent was informed and (2) what type you obtained (for instance, written or verbal, and if verbal, how it was documented and witnessed). If your study included minors, state whether you obtained consent from parents or guardians. If the need for consent was waived by the ethics committee, please include this information.
If you are reporting a retrospective study of medical records or archived samples, please ensure that you have discussed whether all data were fully anonymized before you accessed them and/or whether the IRB or ethics committee waived the requirement for informed consent. If patients provided informed written consent to have data from their medical records used in research, please include this information.

AUTHORS: We have updated this section appropriately.

3. Please update your submission to use the PLOS LaTeX template. The template and more information on our requirements for LaTeX submissions can be found at http://journals.plos.org/plosone/s/latex.

AUTHORS: We have reviewed the guidelines and updated the body of text accordingly.

4. We note that the grant information you provided in the ‘Funding Information’ and ‘Financial Disclosure’ sections do not match.
When you resubmit, please ensure that you provide the correct grant numbers for the awards you received for your study in the ‘Funding Information’ section.

AUTHORS: We have updated this section appropriately.

5. In your Data Availability statement, you have not specified where the minimal data set underlying the results described in your manuscript can be found. PLOS defines a study's minimal data set as the underlying data used to reach the conclusions drawn in the manuscript and any additional data required to replicate the reported study findings in their entirety. All PLOS journals require that the minimal data set be made fully available. For more information about our data policy, please see http://journals.plos.org/plosone/s/data-availability.

Upon re-submitting your revised manuscript, please upload your study’s minimal underlying data set as either Supporting Information files or to a stable, public repository and include the relevant URLs, DOIs, or accession numbers within your revised cover letter. For a list of acceptable repositories, please see http://journals.plos.org/plosone/s/data-availability#loc-recommended-repositories. Any potentially identifying patient information must be fully anonymized.

AUTHORS: We have created a deidentified dataset to be uploaded that will be uploaded to our supplement in accord with *PLOS ONE*’s policies. Some variables such as gender and race were removed due to internal policies to ensure protection of private health information. These variables can be made available upon author request.
